# Whole genome Shotgun Sequence of *Anopheles stephensi,* The Host of Malaria parasite, *Plasmodium* sp.

**DOI:** 10.7150/jgen.121872

**Published:** 2025-09-22

**Authors:** Jack Farah, Junhyeok Jang, Kenza Madhi, Jessica Phan, Elizabeth Pratt, Amrita Sankrit, Nora L. Sharma, Aria Suchak, Devin W. Thomas, Shimaa M. Ghazal

**Affiliations:** 1 Science Department, Phillips Exeter Academy, Exeter, NH 03833, USA.; 2 Department of Computer science, Kingsbury Hall, University of New Hampshire, Durham, NH, 03824, USA.

**Keywords:** *Anopheles stephensi*, Shotgun sequence, Genome assembly, Malaria, NovaSeq 6000 Illumina, GenBank

## Abstract

*Anopheles stephensi*, one of the main mosquito vectors for malaria in Asia. It belongs to the same complex species of *Anopheles gambiae*. A genome assembly was performed on female *Anopheles stephensi,* the resulting genome was 201Mbp in size and consisted of 32,280 contigs with an N50 of 21,1 kb and a GC content of 45%.

## Introduction

Malaria causes a significant health burden, not only in Africa but around the world. It is a mosquito-borne disease caused by protozoan parasites of the genus *Plasmodium.* In 2022, an estimated 249 million cases and 608,000 deaths were reported worldwide 1. Malaria is transmitted among humans by mosquitoes of the genus *Anopheles,* which deliver parasitic plasmodium from an infected human to a non-infected human 2,3. One such mosquito is *Anopheles stephensi (A. stephensi),* whose genome we have fully sequenced and assembled*.*

*Anopheles stephensi* is a prominent mosquito vector species for *Plasmodium falciparum (P. falciparum)* and *Plasmodium vivax (P. vivax)* malaria 4,5,6. Due to its physiological, behavioral, and genetic similarities with those of fellow mosquito malaria vector *A. gambiae*, *A. stephensi* belongs to the same subgenus as *A. gambiae* 4,6.

A. *stephensi* mosquito life cycles are highly variable dependent on temperature, as complete maturation may occur within 2-3 days at 30°C but take up to 7-14 days at temperatures of 16°C. Temperature and nutrition play a crucial rule in the survival rate of the adult during their larval stage 7,8,10. A relative humidity of 60% and temperatures between 21-32°C create the most conducive conditions for transmission of *Plasmodium* through *A. stephensi.* Geographic locations with these characteristics are natural breeding grounds for *A. stephensi* 9,11,12.

*A. stephensi* is native to Asia but has firmly established itself as an invasive malaria vector in Eastern and Southern Africa. As of 2022, strains of *A. stephensi* are responsible for some malaria infections in five regions of the world: West Africa, East Africa, Southern Africa, the Eastern Mediterranean, and Southeast Asia 1,3,9.

*A. stephensi s.l*. is the specific strain that transports *P. falciparum* in West Africa, and responsible for 100% of malaria human infections in this region, while in East and southern African countries with low rates of malaria transmission, *P. falciparum* is responsible for 91% of malaria infections. The remaining 9% is attributed almost entirely to *P. vivax,* and less than 1% of infections are caused by other plasmodium species 1.

The life cycle of* A. stephensi* includes four life forms: eggs, larval, pupal, and terrestrial. The egg, larval, and pupal stages comprise the aquatic phase of the life cycle, which lasts 5-14 days, depending on the temperature of the mosquito's environment. An *A. stephensi* mosquito enters the terrestrial stage when it matures into an adult 5. The life cycle begins after a female *A. stephensi* ingests a blood meal and lays 50-300 eggs on the surface of a body of water. At 30°C, larvae hatch from these eggs within 2-3 days. They then live on the water's surface, feeding on organic detritus and molting 5,7. After molting 4 times, the larvae transition into the pupal stage. After 2-3 more days, the pupae mature into adults and immediately navigate to sugar sources to ingest necessary nutrients for survival, flight, and reproduction. The adult mosquitoes generally survive up to a month after emergence but can survive for longer under ideal conditions 5,7,12.

## Materials and Methods

Our *Anopheles stephensi* specimens were originally isolated from India, Asia. The female *Anopheles stephensi,* were obtained from Catteruccia Lab at Harvard T. H. Chan School of Public Health, MA, US. DNA extraction was carried out from three adult female mosquitos that didn't receive a blood meal, using the Quick-DNA Tissue/Insect Miniprep Kit (D6016, ZYMO Research, Irvine, California, USA) and following the manufacturer's instructions. This step immediately was followed by library preparation, using Illumina DNA Prep, (M) Tagmentation, Accession number: 20060060), and IDT® for Illumina® DNA/RNA UD Indexes Set A, Tagmentation (Accesion number: 20027213) were used. The library then sent to Hubbard Center for Genome Studies (University of New Hampshire, Durham, NH), where the draft genome of *Anopheles stephensi* was generated using a NovaSeq 6000 (Illumina) with a paired end, version 1.5 chemistry on an SP, patterned flow cell, forward and reverse read lengths were 250 base pairs and indexing reads were dual 8-mers.

## Results

A standard Illumina shotgun library was constructed and sequenced using the Illumina HiSeq 2000 platform, which generated 26,140,783 reads. The Illumina sequence data was demultiplexed using Illumina bcl2fastq v2.20.0.422 (Illumina) and quality checked using FastQC v0.11.5 13, then assembled using MaSuRCA v. 4.1.0 14. The high-quality draft genome of *Anopheles stephensi* was resolved to 32,280 contigs, with an N50 of 21.1 bp with a GC content of 45%. The total size of the genome is 244 Mbp, and the final assembly is based on 201Mb of Illumina draft data, which is provided by an average 45X coverage of the genome.

## Discussion

This Whole Genome Shotgun project has been deposited at DDBJ/ENA/GenBank under the accession JBFRBV000000000. The version described in this paper is version JBFRBV010000000.1 https://www.ncbi.nlm.nih.gov/nuccore/JBFRBV000000000.1.
